# Genomic and transcriptomic characterization of *Pseudomonas aeruginosa* small colony variants derived from a chronic infection model

**DOI:** 10.1099/mgen.0.000262

**Published:** 2019-03-28

**Authors:** Sharon Irvine, Boyke Bunk, Hannah K. Bayes, Cathrin Spröer, James P. R. Connolly, Anne Six, Thomas J. Evans, Andrew J. Roe, Jörg Overmann, Daniel Walker

**Affiliations:** ^1^​ Institute of Infection, Immunity and Inflammation, College of Medical, Veterinary and Life Sciences, University of Glasgow, Glasgow G12 8QQ, UK; ^2^​ Leibniz-Institut DSMZ – Deutsche Sammlung von Mikroorganismen und Zellkulturen, Inhiffenstraße 7B, 38124 Braunschweig, Germany; ^3^​ German Centre of Infection Research (DZIF), Partner site Hannover-Braunschweig, Braunschweig, Germany

**Keywords:** *Pseudomonas aeruginosa*, small colony variants, chronic infection, recombination, transcriptomics

## Abstract

Phenotypic change is a hallmark of bacterial adaptation during chronic infection. In the case of chronic *Pseudomonas aeruginosa* lung infection in patients with cystic fibrosis, well-characterized phenotypic variants include mucoid and small colony variants (SCVs). It has previously been shown that SCVs can be reproducibly isolated from the murine lung following the establishment of chronic infection with mucoid *P. aeruginosa* strain NH57388A. Using a combination of single-molecule real-time (PacBio) and Illumina sequencing we identify a large genomic inversion in the SCV through recombination between homologous regions of two rRNA operons and an associated truncation of one of the 16S rRNA genes and suggest this may be the genetic switch for conversion to the SCV phenotype. This phenotypic conversion is associated with large-scale transcriptional changes distributed throughout the genome. This global rewiring of the cellular transcriptomic output results in changes to normally differentially regulated genes that modulate resistance to oxidative stress, central metabolism and virulence. These changes are of clinical relevance because the appearance of SCVs during chronic infection is associated with declining lung function.

## Data Summary

All genome sequences are deposited in NCBI GenBank under accession numbers CP013477, CP013478 and CP013479. Transcriptome data are deposited at the EMBL-EBI ENA database under study number PRJEB12456.

**Impact Statement**

Chronic lung infection with *Pseudomonas aeruginosa* is the major proven cause of mortality in patients with cystic fibrosis. This is despite the use of aggressive antibiotic therapy, which although effective in managing infection, frequently fails to eradicate *P. aeruginosa*. Adaptation to the lung is associated with the appearance of small colony variants (SCVs), which are highly adherent and adept at forming biofilms. Here we show that SCVs isolated from the murine lung during chronic infection, and in the absence of antibiotic therapy, show transcriptional changes that enhance the oxidative stress response and increase virulence. Sequencing of the genomes of the SCVs and parent strain shows that the SCVs contain a large genomic inversion relative to the parent strain.

## Introduction

Phenotypic variation is a hallmark of adaptation to the host during chronic bacterial infection. There is considerable interest in slow-growing subpopulations of bacteria, termed small colony variants (SCVs), due to their association with persistent infections [1, 2]. The SCV variant is common to diverse bacteria and is characterized by phenotypes including reduced growth, increased biofilm production [3], antibiotic resistance and hyperpiliation. SCVs have been described for a wide range of bacterial genera and species including *Staphylococcus aureus* [4, 5], *Staphylococcus epidermidis* [6], *Streptococcus* sp. [7, 8], *Enterococcus* [9], *Listeria* [10], *Burkholderia* [11], *Salmonella* [12], *Brucella* [13], *Lactobacillus*, *Serratia* and *Neisseria* [14]. In the case of *Pseudomonas aeruginosa*, SCVs are commonly associated with chronic infection of the lung in patients with cystic fibrosis (CF) [15, 16].

*P. aeruginosa* is the major proven cause of mortality in patients with CF and chronic infection leads to a progressive decline in pulmonary function and inevitably respiratory failure [17–19]. Despite intensive anti-pseudomonal chemotherapy greatly improving the prognosis for CF patients [20], the current median age at death for CF patients is around 30 years in developed countries [21]. The frequent failure of antibiotic therapy and host defences to eradicate *P. aeruginosa* from the CF lung is thought to be largely due to the increased antibiotic tolerance when growing in the biofilm state and the appearance of mucoid phenotypic variants that are a hallmark of adaptation in the chronically infected lung. A further complicating factor is the appearance of highly adherent SCVs that are adept at biofilm formation [15, 22, 23]. *P. aeruginosa* SCVs may display high intracellular levels of c-di-GMP [23–27], enhanced biofilm formation, high fimbrial expression, repression of flagellar genes, resistance to phagocytosis and enhanced antibiotic resistance. Most importantly, the appearance of SCVs in the CF lung correlates with poor clinical outcome [11, 28–31].

There are a range of genetic changes that have been shown to be responsible for the phenotypic switch to the SCV phenotype in *P. aeruginosa*, including mutations in the Wsp system and *yfiBNR* operon that form part of the c-di-GMP regulatory system in *P. aeruginosa* [26, 32–34]. However, identification of the major clinically relevant pathways of conversion to the SCV phenotype is complicated by the unstable phenotype displayed by many SCVs with reversion to a normal colony phenotype frequently observed, preventing successful comparative genetic studies on clinical SCVs and their closely related parent strains. In *S. aureus*, which also forms clinically relevant SCVs, recent work has shown that a reversible large-scale chromosomal inversion is the genetic basis of the switch between a normal colony and SCV isolated from the same patient [35]. In addition, *S. aureus* SCVs, which are commonly isolated from the CF lung, are highly resistant to oxidative stress, suggesting that conversion to the SCV phenotype may be an adaptation to the environment in chronically inflamed host tissue [36].

In the present study, we have attempted to determine the genetic basis of phenotypic conversion from the mucoid to the SCV phenotype for SCVs isolated from the chronic lung infection model described by Bayes *et al.* [37]. For two SCVs isolated from this work, we have shown through a combination of single-molecule real-time (SMRT; Pacific Biosciences) and Illumina sequencing that a large and stable chromosomal inversion and associated truncation of a 16S rRNA gene accompanies conversion to the SCV phenotype. The phenotypic switch is characterized by transcriptional changes to a large number of genes that most notably include downregulation of several genes encoding metabolic enzymes, DNA repair proteins and heat shock proteins and upregulation of genes encoding proteins involved in the response to oxidative stress. The absence of other obvious genetic changes suggests that this chromosomal inversion may be the genetic basis of conversion to the SCV phenotype.

## Results

*P. aeruginosa* SCVs are commonly isolated from patients with CF and have been isolated *in vitro* as well as from experimental infection models following aminoglycoside treatment [16, 28]. Bayes *et al.* describe the isolation and partial characterization of SCVs isolated from a chronic murine *P. aeruginosa* lung infection model [37]. In this model, animals were inoculated with *P. aeruginosa* strain NH57388A (NHMuc), a mucoid clinical isolate, embedded in agar beads. NHMuc has a known mutation in the gene encoding the anti-sigma factor MucA, which results in alginate overproduction [38, 39]. Recovered bacteria from lung homogenate samples display two distinct colony morphologies: typical large mucoid colonies identical in morphology to the inoculating strain and SCVs. Mucoid colonies were evident after 24 h of growth on agar plates at 37 °C with SCVs visible only after 48 h of growth on agar plates [37].

To understand the genetic basis of this phenotypic change, we initially performed Illumina HiSeq whole-genome sequencing and genomic comparison between NHMuc and two separate SCVs (SCVJan and SCVFeb) isolated from independent *in vivo* experiments. However, despite their gross phenotypic differences, this analysis failed to identify any genetic differences between the SCVs and the parent strain.

**Fig. 1.**
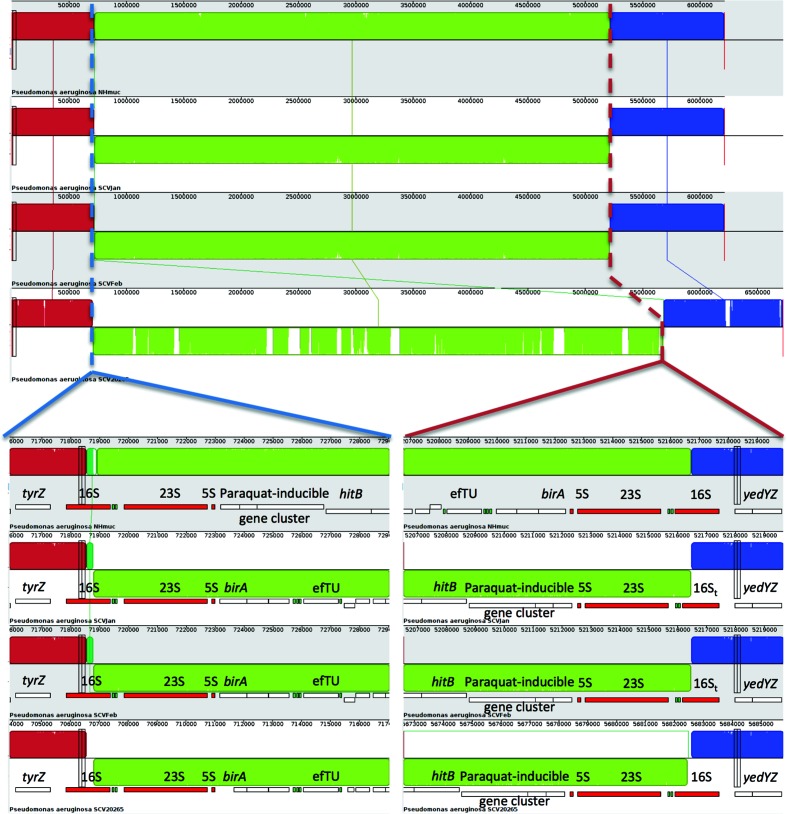
A common large-scale chromosomal inversion in three *P. aeruginosa* strains is the genetic basis for conversion to the SCV phenotype. From top to bottom strains NHMuC, SCVJan, SCVFeb and SCV20265 are displayed. Dashed lines indicate the inversion breakpoints present in the 16S rRNA genes. An inversion with highly similar breakpoints is present in the genome of strain SCV20265, an SCV isolated from a patient with CF. Within strains SCVJan and SCVFeb, a unique truncated version of the 16S rRNA gene (16S_t_) could be resolved, which could not be detected in strain SCV20265.

**Fig. 2.**
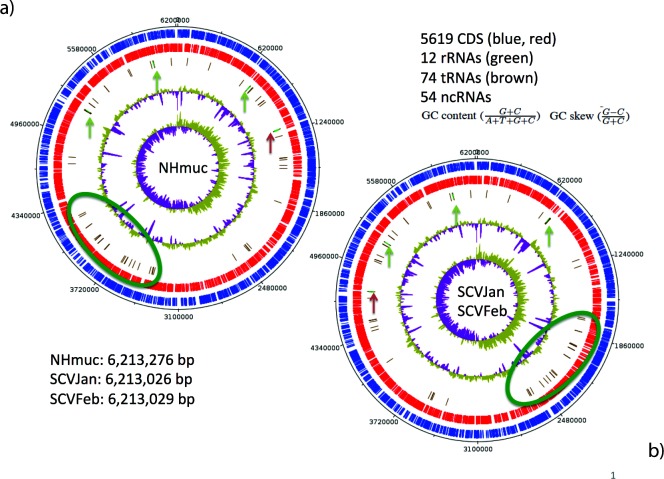
Chromosomal maps of *P. aeruginosa* NHmuc (a) and SCVJan/SCVFeb (b). The circular genomes of both strains are shown. Genomes of both SCV strains are 250 bp smaller than the parental strain NHmuc. Exact genome sizes are given in the lower left corner. In blue (circle 1) genes lying on the forward strand are shown and in red (circle 2) those on the reverse strand. In circle 3 tRNA genes are shown in brown, often clustered together with green rRNA genes, which have been additionally marked by vertical arrows. The red arrow shows the transposition of rRNA operon 3 in addition to that of a large tRNA region (green ellipse) due to the described chromosomal inversion. Circle 4 shows the GC content, whereas in circle 5 a GC skew is shown. Numbers of coding sequences, rRNAs, tRNAs and non-coding RNAs are identical in all strains (upper right corner according to GenBank submission).

Next, we used the ultra-long reads produced by SMRT PacBio sequencing to attempt to identify any large-scale genome rearrangements that could drive conversion to the SCV phenotype. Using this technique we identified a large-scale genomic inversion accompanying conversion from the parent mucoid to SCV phenotype in both SCVJan and SCVFeb. Closer inspection of the genome sequence identified the start and end points of the inversion, which for both SCVJan and SCVFeb begins at the first rRNA operon (0.72 Mbp) and ends at the third rRNA operon (5.21 Mbp). Exact chromosomal breakpoints were identified in the corresponding 16S rRNA genes by performing a mauve breakpoint analysis (Fig. 1). Furthermore, genome analysis revealed a 250 bp shortened 16S rRNA gene (16St) in both SCV strains, which is reflected in the reduced genome sizes of the SCVs (SCVJan 6 213 026 bp, SCVFeb 6 213 029 bp; Fig. 2a) compared to the parent strain, NHmuc (6 213 276 bp; Fig. 2b). There were no further differences in the number of protein coding genes (5619), rRNAs (12) or tRNAs (57) between SCVs and the parent strain. No SNPs could be identified in protein coding genes. A similar genome inversion was not identified (using a PCR-based strategy) in a mucoid strain (NHMucJan) that was phenotypically identical to the parent strain (NHMuc) and isolated from the same chronic infection model as SCVJan (Fig. S1, available in the online version of this article). Interestingly, comparison of the SCVJan and SCVFeb genomes with that of an SCV (SCV20265) isolated from a CF patient [40, 41], which has recently been sequenced by PacBio sequencing, revealed an almost identical chromosomal inversion. However, in SCV20265 the inversion was not accompanied by truncation of the 16S rRNA gene in the third rRNA operon (Fig. 1).

### Transcriptional and phenotypic changes on conversion to the SCV phenotype

**Fig. 3.**
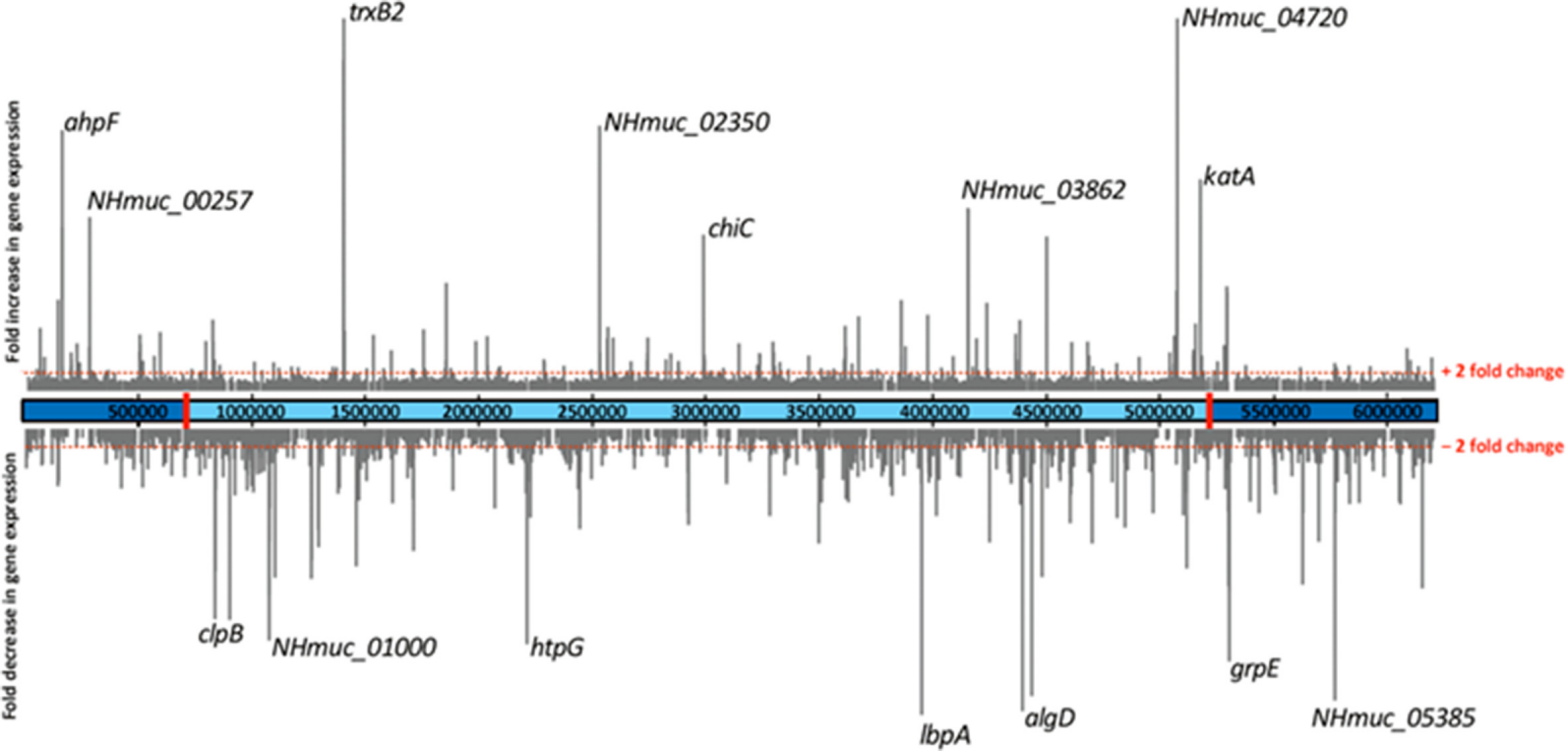
Global changes in gene transcription on conversion to the SCV phenotype. Fold-changes in gene expression for SCVJan/Feb relative to NHMuc are shown in the context of the NHMuc genome. Breakpoints that define the genomic inversion present in SCVJan and SCVFeb are indicated by red rectangles.

To determine the transcriptional changes associated with conversion to the SCV phenotype, we performed RNA sequencing (RNA-Seq) analysis of the parent strain, NHMuc, and two SCV strains grown in Lysogeny broth (LB). Initial analysis showed that SCVJan and SCVFeb have highly similar gene expression profiles that are distinct from that of NHMuc. RNA-Seq data for all strains were collected in triplicate and data for SCVJan and SCVFeb were combined to compare with NHMuc. Relative to NHMuc, 190 genes showed >2-fold upregulation and 364 genes showed >2-fold downregulation in SCVJan/SCVFeb (Table S1). Interestingly, the transcriptional changes associated with genomic inversion and that drive conversion to the SCV phenotype are not restricted to genes close to or within the inversion breakpoints, with major upregulated and downregulated genes distributed relatively evenly throughout the genome (Fig. 3).

**Fig. 4.**
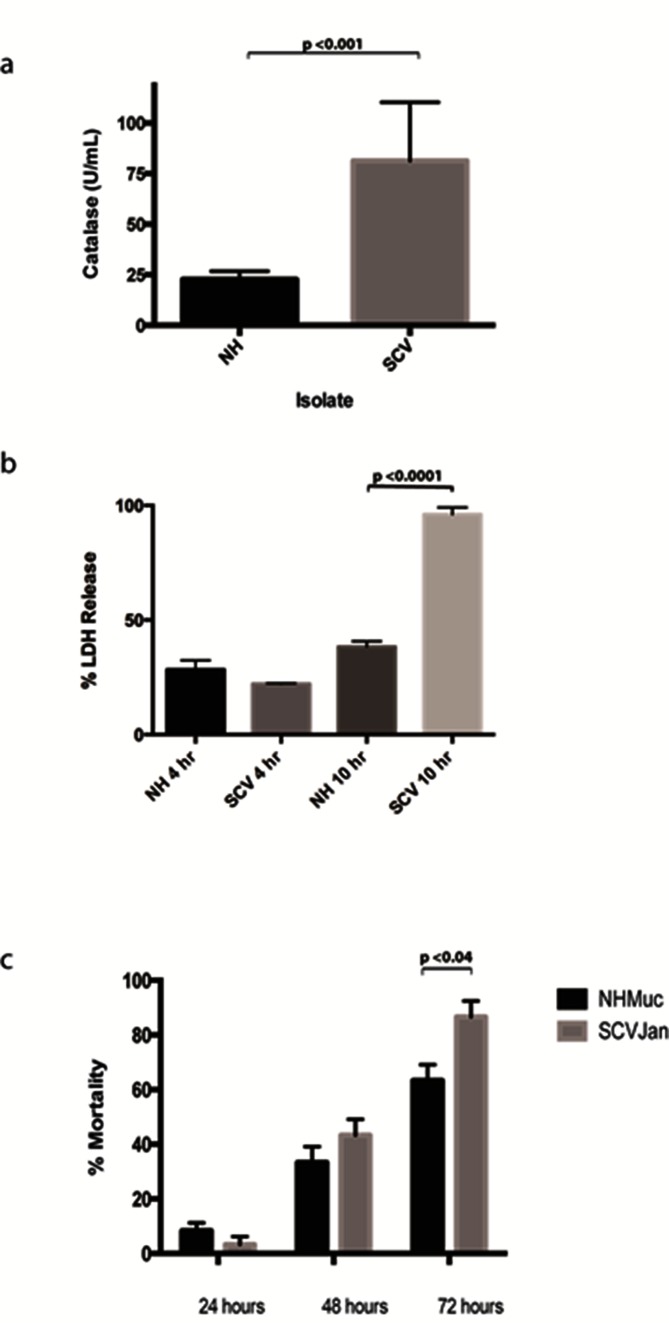
Phenotypic characterization of SCVJan. (a) Catalase activity assay demonstrating a marked increase in catalase activity in the SCV as compared to the NH parent strain. (b) Percentage lactate dehydrogenase (LDH) released from a macrophage cell line with comparison between NHMuc and SCVJan over a 4 and 10 h time period. (c) *Galleria mellonella* larvae survival over time when infected with the NHMuc and SCVJan strains monitored over 72 h.

Major functional classes of genes downregulated in SCVJan/SCVFeb include those involved in energy metabolism, amino acid and protein biosynthesis, DNA replication and recombination, and cell wall/lipopolysaccharide/capsule biosynthesis, which together are consistent with the slow growth rate observed for SCVs. Notably, genes encoding heat shock proteins and other molecular chaperones (IbpA, GrpE, HtpG, ClpB, DnaK, GroES, DnaJ and ClpX) are highly represented among the most strongly downregulated genes in the SCVs (Table 1). Conversely, genes that function in the response to oxidative stress and those that encode secreted virulence factors are largely upregulated in SCVJan/SCVFeb. Indeed, five of the ten most highly upregulated genes in SCVJan/SCVFeb are those associated with the response to oxidative stress (Table 2). Highly upregulated oxidative stress genes include *katA* [41], which encodes the major catalase of *P. aeruginosa*, *ahpB*, *ahpC* and *ahpF* [42, 43], which encode subunits of alkyl hydroperoxide reductase, and *trxB2*, which encodes thioredoxin reductase 2 [44, 45]. Consistent with the observed transcriptional changes, catalase activity was strongly increased in SCVJan relative to NHMuc (Fig. 4a).

**Table 1.** Highly downregulated genes in SCVs relative to NHMuc

**Gene ID**	Fold change*	***P***†	**Protein description**	**PAO1 gene ID**
NHmuc_01025	−75	2.6×10^−11^	Putative acetyltransferase	PA4364
mexC	−53	1.1×10^−11^	RND multidrug efflux fusion protein MexC	PA4599
ibpA	−31	1.1×10^−7^	Heat-shock protein IbpA	PA3126
algD	−30	1.0×10^−9^	GDP-mannose 6-dehydrogenase AlgD	PA3540
NHmuc_05385	−29	8.5×10^−8^	17 kDa surface antigen	PA5182
NHmuc_04138	−29	1.3×10^−10^	Periplasmic metal-binding protein	PA3520
grpE	−25	1.2×10^−7^	Heat shock protein GrpE	PA4762
htpG	−23	3.6×10^−7^	Heat shock protein 90	PA1596
NHmuc_0100	−23	1.8×10^−5^	Hypothetical, unclassified, unknown	
clpB	−21	6.5×10^−6^	ClpB protein	PA4542
nfxB	−21	3.7×10^−7^	Transcriptional regulator NfxB	PA4600
fsxA	−18	7.9×10^−8^	FxsA protein	PA4387
NHmuc_04950	−18	3.3×10^−6^	Molecular chaperone DnaK	PA4761
NHmuc_05744	−17	5.8×10^−5^	Putative lipoprotein	PA5526
dapB	−17	2.8×10^−6^	Dihydrodipicolinate reductase	PA4759
hslV	−17	4.1×10^−7^	ATP-dependent protease subunit	PA5053
NHmuc_01173	−16	4.7×10^−6^	Hypothetical protein	PA2756
NHmuc_01024	−16	6.1×10^−7^	Putative transporter	PA4365
NHmuc_04180	−16	3.3×10^−6^	Recombinase A	PA3617
mexD	−15	9.5×10^−7^	RND multidrug efflux transporter MexD	PA4598
NHmuc_04776	−15	6.1×10^−6^	PAS/PAC sensor signal transduction histidine kinase	PA4197
rsmA	−15	7.5×10^−4^	Carbon storage regulator	PA0905
NHmuc_01174	−14	3.3×10^−7^	Hypothetical protein	PA0737
NHmuc_01594	−13	2.1×10^−5^	Putative oxidoreductase	PA1137
mucA	−13	7.4×10^−5^	Anti-sigma factor MucA	PA0763
NHmuc_03262	−12	2.5×10^−9^	Hypothetical protein	PA3505
NHmuc_04386	−12	6.8×10^−5^	Surface antigen	PA3819
amrZ	−12	3.0×10^−5^	Alginate and motility regulator Z	PA3385
glnA	−12	5.8×10^−6^	Gutamine synthetase	PA5119
groES	−12	3.9×10^−5^	Co-chaperonin GroES	PA4386
dnaJ	−12	7.5×10^−5^	Chaperone protein DnaJ	PA4760
algU	−11	1.8×10^−4^	RNA polymerase sigma factor AlgU	PA0762
clpX	−11	4.5×10^−4^	ATP-dependent protease subunit ClpX	PA1802
NHmuc_04521	−11	1.9×10^−6^	Periplasmic ligand-binding sensor	PA3952

*The magnitude of gene expression (fold change) was determined by comparing transcription in three replicates of NH with that in three replicates each of the two SCV strains.

†*P*-values were assessed by performing an EDGE test using CLC software.

**Table 2.** Highly upregulated genes in SCVs relative to NHMuc

**Gene ID**	Fold change*	***P*†**	**Protein description**
NHmuc_04720	62	3.2×10^−16^	Hypothetical protein
trxB2	55	4.0×10^−16^	Thioredoxin reductase 2
NHmuc_01300	35	3.7×10^−15^	Putative alkyl hydroperoxide reductase
NHmuc_02350	28	1.4×10^−26^	Putative acyl carrier protein
ahpF	28	8.4×10^−13^	Alkyl hydroperoxide reductase subunit F
kata	23	7.1×10^−10^	Catalase
NHmuc_03862	20	6.9×10^−21^	Putative ankyrin domain-containing protein
NHmuc_03863	19	1.8×10^−18^	Putative hydrolase
NHmuc_00257	19	1.4×10^−12^	Putative CBS domain protein
ahpC	18	9.0×10^−8^	Alkyl hydroperoxide reductase subunit C
chiC	17	1.5×10^−9^	Chitinase
NHmuc_04185	17	6.7×10^−6^	RNA polymerase sigma factor RpoS
aprA	12	5.1×10^−8^	Alkaline metalloproteinase
NHmuc_04924	11	3.2×10^−16^	CsbD family protein
NHmuc_04718	11	1.5×10^−11^	Hypothetical protein
NHmuc_04925	11	2.2×10^−8^	Transport-associated
NHmuc_00127	10	5.9×10^−7^	Putative haemolysin
Snr1	10	4.9×10^−7^	Cytochrome c Snr1
lecB	9	5.9×10^−8^	Fucose-binding lectin PA-IIL
NHmuc_03413	8	9.1×10^−15^	Phage terminase, small subunit
katB	8	9.1×10^−6^	Catalase
NHmuc_04074	8	2.2×10^−12^	Leucyl-tRNA synthetase
phzG2_2	7	3.3×10^−6^	Pyridoxamine 5′-phosphate oxidase
NHmuc_03358	7	2.0×10^−8^	Putative protein associated inclusion bodies
phzE1_1	7	3.2×10^−6^	Phenazine biosynthesis protein PhzE
NHmuc_00055	7	6.1×10^−7^	Hypothetical protein
NHmuc_01628	7	1.3×10^−8^	Hypothetical protein
cbpD	7	4.1×10^−6^	Chitin-binding protein CbpD
NHmuc_00546	6	2.0×10^−3^	LysR transcriptional regulator
rhlR	6	1.2×10^−4^	Transcriptional regulator RhlR
gcdH	6	4.3×10^−5^	Glutaryl-CoA dehydrogenase
NHmuc_01422	6	4.1×10^−4^	Putative DNA-binding stress protein
NHmuc_04078	6	1.5×10^−10^	Oxidoreductase probably involved in sulfite reduction
rsaL	6	1.2×10^−2^	Regulatory protein RsaL

*The magnitude of gene expression (fold change) was determined by comparing transcription in three replicates of NH with that in three replicates each of the two SCV strains.

†*P*-values were assessed by performing an EDGE test using CLC software.

Genes encoding a number of secreted virulence factors such as the proteases LasA, LasB and AprA, the frucose-binding lectin LecB [46] and the chitin binding protein CbpD and chitinase ChiC [47] were also highly upregulated. Similarly, genes encoding hydrogen cyanide synthase and a number of enzymes that function in phenazine biosynthesis are also upregulated (Table S1) [25, 48–51]. Phenazines have previously been shown to enhance killing of *Caenorhabditis elegans* by *P. aeruginosa* [52]. The apparent increase in the production of virulence factors by SCVJan/SCVFeb relative to NHMuc suggests increased virulence of the SCV. To directly test this we used an infection model based on infection of the murine macrophage cell line J774A.1. Cell death of J774A.1 through lactate dehydrogenase (LDH) release was measured 4 and 10 h after infection with NHMuc and SCVJan. At 4 h, levels of LDH release were similar for NHMuc and SCVJan, whereas at 10 h LDH release was significantly increased for SCVJan (96 vs 38 %, *P*<0.0001; Fig. 4b). To determine if the increased virulence of the SCV observed against a murine cell line translated to increased virulence in an animal model of infection, we used an invertebrate model of infection using the larva of the wax moth *Galleria mellonella*. Similar to the macrophage infection assay, SCVJan displayed increased virulence in the *G. mellonella* infection model. Mortality of larvae was measured 24, 48 and 72 h after infection. No significant differences in mortality were detected at 24 or 48 h, whereas at 72 h mortality was 86 and 63 % (*P*<0.04) for SCVJan- and NHMuc-infected larvae, respectively (Fig. 4c). Data from both infection models indicate that SCVJan shows increased virulence, relative to NHMuc, which is consistent with the phenotype of SCVs obtained from the human host [29, 53].

## Discussion

In this work we show that *P. aeruginosa* SCVs isolated from a chronic murine lung infection model display a general upregulation of virulence-associated genes, relative to the mucoid parent strain, and increased virulence, which may begin to explain the link between the appearance of SCVs in chronic lung infection and the associated decline in lung function [54]. In addition, the immediate upregulation of genes that mediate the response to oxidative stress suggests why the isolated SCVs are rapidly selected for in a chronic infection model in which the host immune system is strongly activated.

A key strength of our study was the availability of both the parent strain used to establish infection in a chronic infection model and the derived SCVs that evolved during infection. This allowed for a meaningful comparative genetic analysis to be performed to probe the genetic changes that occurred on conversion to the SCV phenotype. Surprisingly, SNPs and short insertions and deletions (indels) were not identified in the SCV genome by Illumina sequencing, and SMRT sequencing was subsequently used to show that the two sequenced SCVs carried a large genomic inversion within 16S rRNA genes. Interestingly, the transcriptional changes associated with genomic inversion are not restricted to genes close to or within the inversion breakpoints, but distributed relatively evenly throughout the genome. Instead the major changes in gene expression are largely restricted to specific functional classes of genes including those that mediate the response to oxidative stress, virulence, DNA repair and recombination, the chaperone network and metabolism. This global rewiring of the cellular transcriptomic output results in concerted transcriptional changes to these normally differentially regulated genes. However, the mechanisms that underlie these transcriptional changes are not clear. A study in *Escherichia coli* suggested that positional effects on gene expression may be due to local differences in chromosomal structuring and organization, with DNA gyrase playing an important role at certain high-activity locations [55]. Further studies will be needed to clarify such positional effects on gene expression in the SCV studied here.

Other large-scale genome rearrangements including large chromosomal inversions have previously been described in *P. aeruginosa*, but these were not associated with conversion to the SCV phenotype [56]. A reversible genomic inversion has also recently been shown to mediate the reversible conversion between normal colony and SCV phenotypes in *S. aureus* [35]. However, in the case of the SCVs isolated in our work, the SCV phenotype is stable and revertants to the parent phenotype were not observed. A possible explanation for this observation is that a number of genes encoding proteins involved in DNA repair and recombination, including RecA, are downregulated in the SCV relative to the parent strain (Table 1).

In conclusion, we have shown that a *P. aeruginosa* SCV that originated in the lungs of an animal with chronic colonization may result from a large chromosomal inversion and associated truncation of an rRNA gene. Conversion to the SCV phenotype was associated with large-scale transcriptional changes and increased virulence.

## Methods

### Genome assembly and annotation

Purified bacterial genomic DNA was prepared for sequencing on an Illumina HiSeq using Qiagen DNeasy Blood and Tissue Kit as per the manufacturer's protocol. Sequencing and initial bioinformatics were performed in the Centre for Genomic Research, University of Liverpool. Sequencing reads were mapped to the corresponding reference genome (annotated NH strain). SMRTbell template libraries were prepared according to the instructions from Pacific Biosciences, following the Procedure and Checklist Greater than 10 kb Template Preparation and Sequencing. Briefly, for preparation of 10 kb libraries, ~10 µg of genomic DNA isolated from SCVJan, SCVFeb and NHmuc was sheared using g-tubes from Covaris according to the manufacturer's instructions. In total, 5–10 µg of sheared genomic DNA was end-repaired and ligated overnight to hairpin adapters applying components from the DNA/Polymerase Binding Kit P4 from Pacific Biosciences. Reactions were carried out according to the manufacturer′s instructions. SMRTbell template was treated with exonuclease for removal of incompletely formed reaction products. Conditions for annealing of sequencing primers and binding of polymerase to purified SMRTbell template were assessed with the Calculator in RS Remote (Pacific Biosciences). SMRT sequencing was carried out on the PacBio RSII (Pacific Biosciences) taking one 180 min movie for each SMRT cell. In total six, six and five SMRT cells were run respectively. Data from each SMRT cell were assembled independently using the ‘RS_HGAP_Assembly.3’ protocol included in SMRTPortal version 2.3.0 using default parameters. Each assembly revealed the fully resolved chromosome in one single contig. Each chromosome was circularized independently; in particular, artificial redundancies at the ends of the contigs were removed and all chromosomes were additionally adjusted to *dnaA* as the first gene. The validity of each assembly was checked using the ‘RS_Bridgemapper.1’ protocol. For the purpose of this study, it has been confirmed for each of the (repetitive) rRNA operons that enough uniquely mapping long read exists spanning the whole repeat structure. Finally, each genome was error-corrected by a mapping of Illumina reads (paired-end reads, 100 bp) onto finished genomes using Burrows–Wheeler Transform (BWA) [57] with subsequent variant calling using VarScan [58]. A consensus concordance of QV60 could be confirmed for all of the three genomes. Finally, all genomes were annotated using Prokka 1.8 [59]. All genome sequences were deposited in NCBI GenBank under accession numbers CP013477, CP013478 and CP013479. Illumina short read data have been deposited at the EMBL-EBI ENA database under study number PRJEB12456. The shortened 16S rRNA gene for strains SCVJan and SCVFeb was confirmed by PacBio assembly as well as BWA mapping of Illumina reads against the final chromosome showing uniquely mapped reads only at that genome position (data not shown). Genome maps were created using DNAplotter65 [60].

### Transcriptome analysis

RNA isolation of the samples was performed in triplicate. Bacterial suspensions were grown to early stationary phase to an OD_600_ of 1.8 in LB at 37 °C in a shaking incubator. Then, 2 ml of each suspension was pelleted at 12 000 ***g*** for 10 min. RNA was extracted from samples using a bead beating/chloroform extraction method as previously described [61]. The samples were digested with DNAse I for 1 h. Bacterial RNA was enriched using MICROBEnrich (Life Technologies) as per the suggested protocol. rRNA was depleted using Ribo-Zero Magnetic Gold Kit (Epidemiology; Epicentre) as per the manufacturer's protocol. The precipitated sample was resuspended in 20 µl of RNAse-free water. The concentration of RNA was initially determined using Nanodrop followed by an Agilent Bioanalyser. cDNA was generated by using the methods from the Superscript Double-Stranded cDNA Synthesis Kit (Invitrogen) as per the manufacturer’s instructions.

Transcriptome analysis was performed using CLC workbench version 7.0 and significantly upregulated and downregulated genes in SCVJan/SCVFeb versus NHMuc were identified using the CLC software package. Transcriptome data were deposited at the EMBL-EBI ENA database under study number PRJEB12456.

### PCR

Genomic DNA was extracted from 1.5 ml of bacterial culture using the GenElute Bacterial Genomic DNA Kit (Sigma). Extraction was performed following the manufacturer's recommendations and DNA was eluted into 100 µl of Elute Solution. PCR detection of the inversion was performed with KAPA polymerase (KAPA Long-range HotStart PCR Kit; KAPA Biosystems) following the manufacturer’s protocol. Two sets of primers were used: birA-F/yedZ-R and birA-F/tyrZ-R (birA-F: CTCACCGGAGTGGAATC, yedZ-R: TGAGCGCTTACTGCGTGTTCATCCTGG and tyrZ-R: CCATACCGTGCTTATTAATAAGC) with the genomic DNAs of the mucoid and SCV strains recovered from animals amplifying a fragment of 6262 or 6960 bp, respectively.

### *Galleria mellonella* infection model

Larvae were stored on wood chips at 4 °C. Overnight cultures of bacterial strains were grown in LB, diluted 1 : 100 in the same medium and grown to an OD_600_ of 0.3–0.4 as previously described [62, 63]. Cultures were centrifuged and pellets were washed twice and resuspended in 10 mM PBS to an OD_600_ of 0.1. Serial 10-fold dilutions were made in PBS. Five-microlitre aliquots of the serial dilutions were injected using a Hamilton syringe into *G. mellonella* larvae, via the hindmost left proleg as previously described [64]. Ten larvae were injected per dilution for each *Pseudomonas* strain tested. Larvae were incubated in 10 cm plates at 37 °C and the number of dead larvae was scored 1–4 days after infection. For each strain, data from three independent experiments were combined. Larvae were considered dead when they displayed no movement in response to touch. A negative control was used in each experiment to monitor the killing due to physical injury or infection by pathogenic contaminants. Time to death was monitored every 24 h after infection. In any instance where more than one control larvae died in any given experiment, the data from infected larvae were not used.

### LDH release/cytotoxicity assay

To investigate the effect of the *P. aeruginosa* strains on macrophages, we infected the J774A.1 cells with NH and SCV. Bacteria were grown for 17 h to stationary phase in LB at 37 °C. Immediately prior to infection, the bacteria were diluted to exponential growth phase with culture medium lacking phenol red and the concentration was determined by measuring the optical density at 600 nm. Cells were grown, washed and infected as previously documented [65]. Cells were infected with test organisms and incubated for 4 and 10 h. LDH release was determined using the Cytotox 96 cytotoxicity assay kit (Promega) as per the manufacturer’s protocol.

### Catalase activity assay

Overnight cultures of bacterial strains were grown in LB, diluted 1 : 100 in the same medium and grown to an OD_600_ of 0.4. Catalase standards were prepared as per the manufacturer's protocol using the OxiSelect Catalase Activity Assay Kit, Colorimetric (Cell Biolabs). In total, 20 µl of each serial dilution of overnight culture was added to three wells in a 96-well plate to allow for average readings for each sample. Plate absorbance was read at 520 nm using a FLUOstar Optima plate reader (BMG UK).

### Accessibility of biological resources

SCVs used in this study have been deposited at DSMZ under DSM 100776–100778.

## Data Bibliography

1. Eckweiler D, Bunk B, Spröer C, Overmann J, Häussler S. Complete Genome Sequence of Small-Colony Variant SCV20265. DDBJ/EMBL/GenBank no. CP.

## Supplementary Data

Supplementary File 1

Supplementary File 2
