# Supplementary material for: Genomic and transcriptomic characterization of Pseudomonas aeruginosa small colony variants derived from a chronic infection model
Source: Microb Genom. 2019 Mar 28;5(4):e000262. doi: 10.1099/mgen.0.000262 (PMC6521587; doi:10.1099/mgen.0.000262)
Supplement: Supplementary File 1 [file mgen-5-262-s001.pdf]

Supplementary Figure

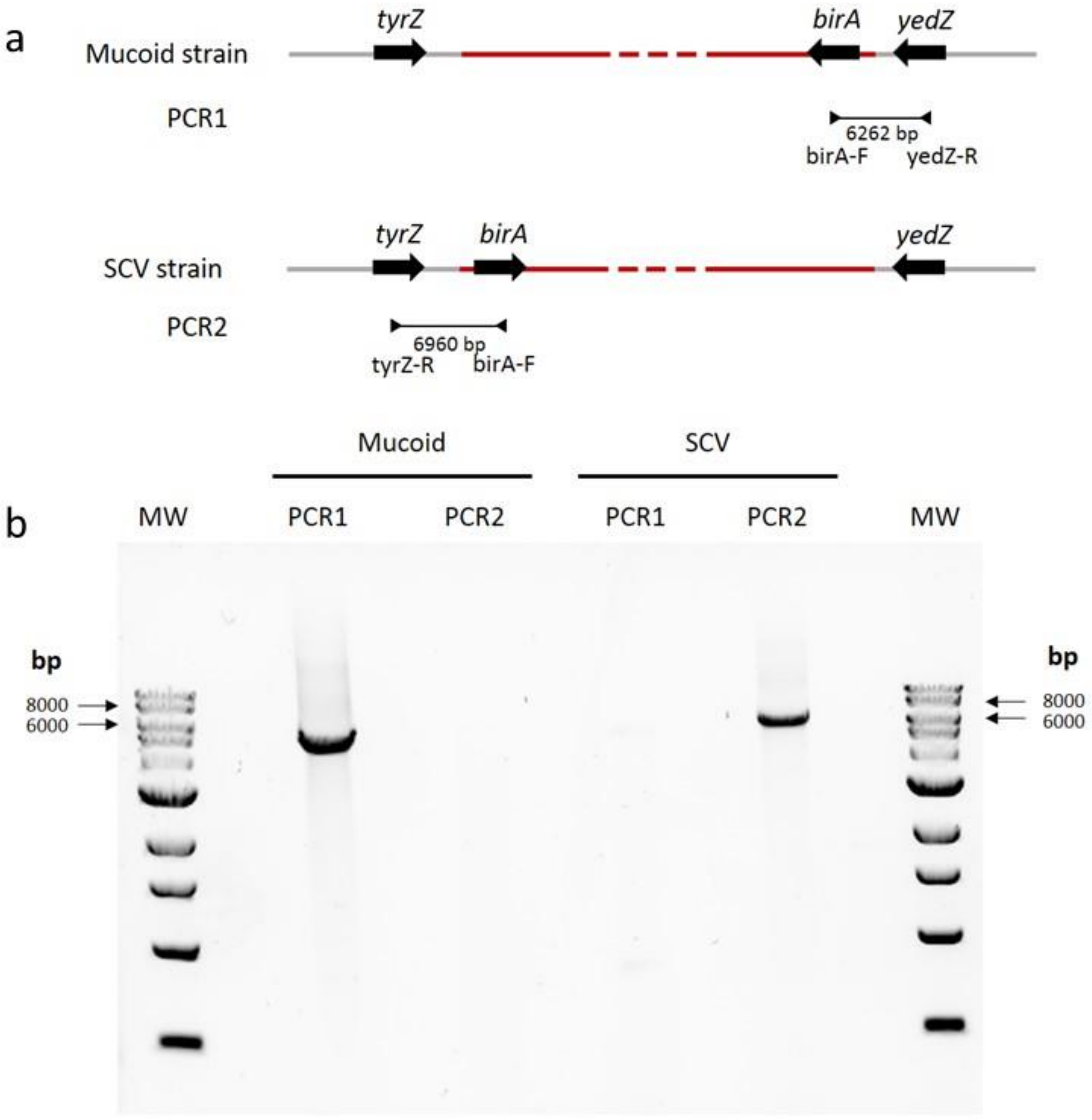

Figure S1. The mucoid strain, NHMucJan, isolated from the same chronic infection model as SCVJan does not contain a similar genome inversion. a) To determine if NHMucJan contains a similar genome inversion as SCVJan and SCVFeb we developed a PCR strategy using primers specific to *tyrZ* or *yedZ* which lie outside and adjacent to the inverted region in combination with primer specific to *birA*, which is adjacent to the rRNA genes within the inverted region. Only the primer pair expected to give a PCR product for each strain is shown. The inverted sequence in the SCV strain is highlighted in red. b) Consistent with the hypothesis that genome inversion drives conversion we observed a PCR product only with birA-F/yedZ-R primer pair for NHMucJan indicating that the genome structure in this region is identical to the parent strain NHMuc.
